# Oral health knowledge of Spanish breastfeeding mothers

**DOI:** 10.1007/s40368-022-00769-9

**Published:** 2022-11-28

**Authors:** L. García- Navas Fernández de la Puebla, M. F. Riolobos González, L. Chico Hernández, C. López Moreno, A. Álvarez Alonso, A. de la Cuesta Aubert, A. Reyes Ortiz

**Affiliations:** 1 Master’s Degree in Paediatric Dentistry Alfonso X El Sabio University, C/Emilio Muñoz 13, 28037 Madrid, Spain; 2 DDS Complutense University of Madrid, Madrid, Spain; 3 Majadahonda, Madrid, Spain

**Keywords:** Pregnancy, Oral health, Breastfeeding

## Abstract

**Introduction:**

The aim of this study is to evaluate the knowledge of infant oral health in mothers who have breastfeeding for a period longer than 6 months.

**Methods:**

A descriptive, observational and cross-sectional study was carried out in 1126 mothers who had breastfed for more than 6 months, using a validated questionnaire.

**Results:**

57% of the mothers surveyed had a high or very high level of knowledge about oral health during pregnancy and breastfeeding; a statistically significant association (*p* < 0.05) was found between this and the variables of mother's age, duration of breastfeeding, level of education and previous information received about oral health.

**Conclusions:**

The age of the mothers is positively related to the level of oral health knowledge, the elder the mothers the higher the knowledge. Mothers with a longer duration of breastfeeding beyond 24 months have a higher degree of oral health knowledge compared to the rest of the respondents, while their level of educations was also positively related to their degree of knowledge. There is a direct relationship between mothers having received previous information on oral hygiene and their surveyed degree of knowledge.

**Supplementary Information:**

The online version contains supplementary material available at 10.1007/s40368-022-00769-9.

## Introduction

Breastfeeding brings enormous benefits for both the child and the mother; it is beneficial in terms of nutrition and prevention of obesity. It is usually at the ideal temperature, reduces economic costs, in addition to improving the mother–child relationship. As for the mother, benefits such as reduced risk of breast and ovarian cancer and type II diabetes have been already described (Henríquez 2010).

The World Health Organization (WHO) and the United Nations International Children's Emergency Fund (UNICEF) recommend initiating breastfeeding within the first hour of life and continuing exclusive breastfeeding for at least the first 6 months of life, supplemented with other foods until at least 2 years of age. WHO has recently launched a global campaign aimed at implementing a maternal and child nutrition plan that includes 6 nutrition targets, including increasing exclusive breastfeeding rates at 6 months of age to 50% of children. One of the main factors associated with the premature abandonment of breastfeeding is insufficient knowledge about breastfeeding. In Spain, there are support groups that can be found on the website of the IHAN (Initiative for the Humanization of Birth and Breastfeeding Care) that provide more information on breastfeeding (Breastfeeding Committee of the Spanish Association of Pediatrics and AEPED).

The advantages of breastfeeding at the myoskeletal level are very notable, increasing mandibular development and advancement, and avoiding atypical swallowing thanks to the toning and correct position of the tongue. In addition, it reduces non-nutritive sucking, both digital and pacifier, and facilitates nasal breathing. As a result, malocclusions such as open bite, anterior and posterior crossbite, maxillary compression, dental crowding and ogival palate, among others, are prevented (Díaz-Gómez et al. 2016).

However, there is controversy about the cariogenicity of breast milk. In the literature we find publications in which prolonged and excessive breastfeeding is associated with caries patterns in early childhood. Demonstrating a causal relationship is complicated by concomitant factors such as bacterial colonization, enamel hypoplasia, sugar intake (contact time of a sugary liquid in the mouth and frequency of intake) and the use of fluorides (Díaz-Gomez et al. 2016).

According to the American Academy of Pediatric Dentistry (AAPD), health protocols that discourage prolonged breastfeeding to reduce the risk of infant caries are not consistent.

The Breastfeeding Committee of the Spanish Association of Pediatrics recommend public discourse on speaking "breastfeeding", patients, and doctors to speak more about it, and proposes as a goal to normalize breastfeeding in children older than 1 year. It is also known that the arrival of primary dentition in children normally produces a decrease in infant sucking, thus reducing the mother's milk production (AEPED; Díaz-Gómez et al. 2016; Ruiz et al. 2014; Salone et al. 2013).

The aim of the study is to assess the knowledge of breastfeeding mothers about the oral health that their children should have, taking into consideration the mother's age, the duration of breastfeeding, the mother's level of education and the mother's previous information about oral health.

## Materials and methods

A cross-sectional, observational and descriptive study was carried out. A questionnaire was designed, previously validated by means of the Aiken V coefficient, consisting of 24 questions, with 3–4 response options. It was divided into 6 sections on oral health knowledge, starting with: dental formation and eruption; clinical etiology and consequences of caries; causes of malocclusion; pediatric dental care; prevention and hygiene; and finally dietary guidelines.

The questionnaire was distributed and performed using the Google Forms^®^ tool to breastfeeding support associations throughout Spain and through various groups of midwives, whose collaboration had been requested previously, and who were responsible for contacting the participants by e-mail. The test was anonymous, in which the objective of the study was reported, and participation was agreed by means of an informed consent form.

As inclusion criteria, only mothers who had been breastfeeding for more than 6 months and currently breastfeeding, and who had completed the questionnaires participated, regardless of the age of the children.

The questionnaire was active in the Google Forms^®^ application for 9 days. A total of 1126 questionnaires registered in the application were obtained as the final sample that met the inclusion criteria.

The qualitative and quantitative variables recorded were mother's age, duration of breastfeeding, mother's level of education, previous information received, and the source of information received, all compared with the mother's level of knowledge.

Using the Google Forms^®^ application, a score of 1 point was assigned to each correct answer and 0 points to each incorrect answer.

Four levels of knowledge were defined: Low (less than 12 points), Regular (13–16 points), High (16–20 points) and Very High (20–24 points) (Cupé-Araujo and García-Rupaya 2015).

The SPSS^®^ version 19.0 statistical package was used for data analysis. The relationships between the results obtained and the different variables were analyzed using Pearson's correlation coefficient, studying to what extent they are or are not independent of each other; the data were presented in contingency tables. A statistical significance value of 0.05 was taken in all cases.

Reliability of the questionnaire: the reliability of the questionnaire was assessed using two different approaches:

- Temporal stability: to determine the stability of the questionnaire over time, the questionnaire was administered twice to a sample of 20 long-term breastfeeding mothers. The interval between surveys was 3 weeks. Once the two sets of results were obtained, the test–retest reliability coefficient was calculated using a Pearson correlation. The calculation of this correlation resulted in *r*_*xy*_ = 0.891, which corresponds to an acceptable reliability *r*_*xx*_ > 0.70 (Arribas 2004).
- Internal consistency: in the present study it was considered appropriate to use the KR20 formula. When calculating the index, a value of KR20 = 0.884 was obtained, which corresponds to a result of internal consistency considered as Good (Arribas 2004).

## Results

A cross-sectional, observational and descriptive study was performed. The average (global) score was 16 correct answers out of 24.

A statistically significant association (*p* < 0.05) was found between level of knowledge about oral health and mother's age, duration of breastfeeding, level of education, and previous information received about oral health. No statistically significant association (*p* > 0.05) was found between level of knowledge and number of children, employment status, marital status and residential environment (rural or urban).

Regarding the Level of Knowledge, 18.8% of the mothers surveyed scored Low, 23.9% Acceptable, 34.2% High and 23.1% Very High (*p* < 0.05) (Table 1).

**Table 1:** Table of categories for the level of knowledge with corrected result

Category	Corrected result	*N*	%
Low	*R*_corr_ < 12	212	18.8
Acceptable	12 > *R*_corr_ ≤ 16	269	23.9
High	17 > *R*_corr_ ≤ 20	385	34.2
Very high	21 ≥ *R*_corr_	260	23.1
Total answers		1126	100

When observing the variable "Mother's age", the age bracket with the largest sample size corresponds to the 30–40 years old group (78.3%). For this group, the High level of knowledge obtained the highest score (27.4%) (Table 1).

**Table 2:**
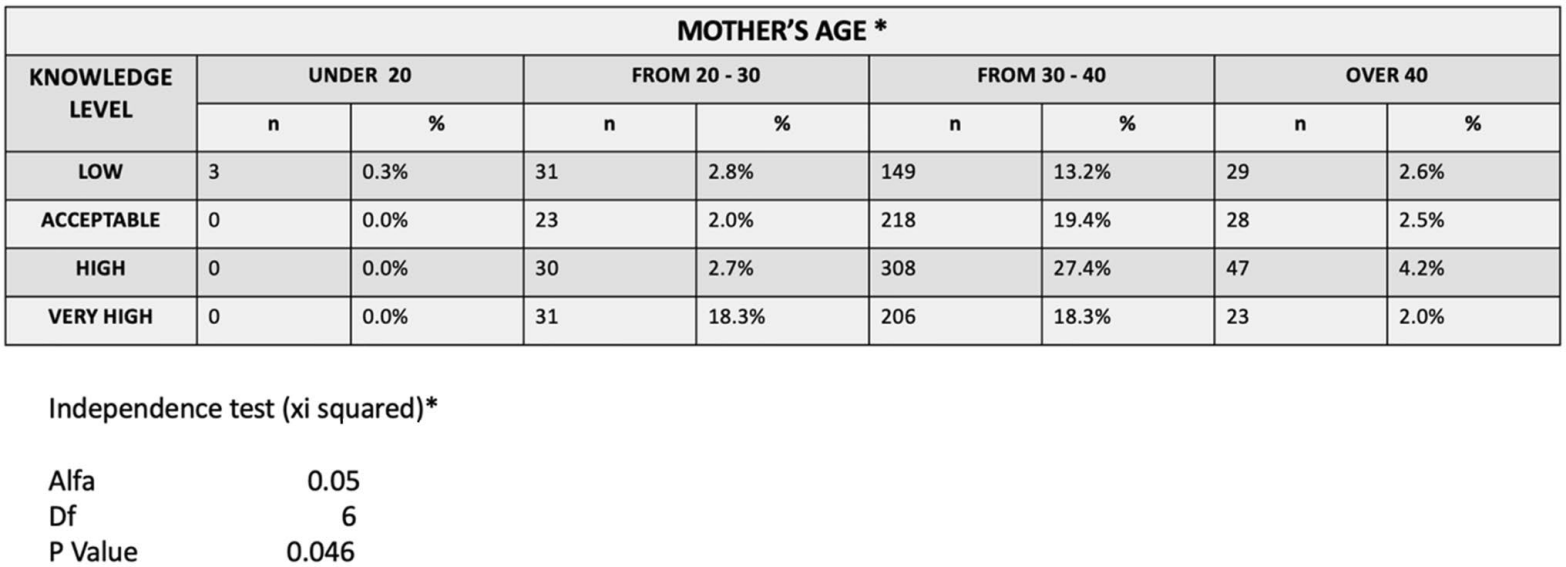
Level of knowledge according to mothers age

In regard to the variable “Duration of breastfeeding”, 60.7% of the questionnaires were completed by mothers who had breastfed for more than 24 months, with the children actually. 27.6% maintained breastfeeding for a period of 12–24 months, and 11.7% lasted between 6 and 12 months of infant age (*p* < 0.05) (Table 2).

**Table 3:**
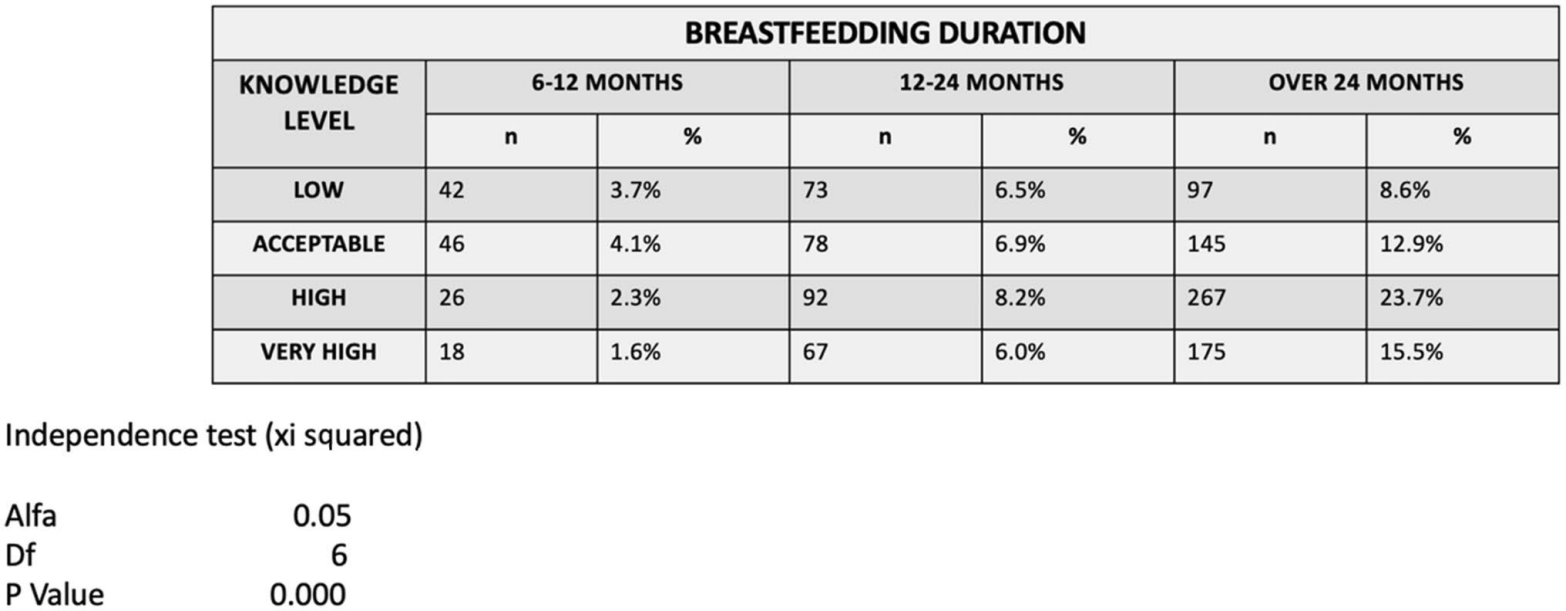
Mother's knowledge level according to breastfeeding duration

Regarding the duration of breastfeeding, the performed statistical test indicates that 66.6% of mothers whose breastfeeding lasts between 6 and 12 months have a low and acceptable knowledge level 7.8%. Mothers whose breastfeeding lasts between 12 and 24 months have very similar results for the 4 levels of knowledge. Finally, 39.2% mothers whose breastfeeding extends beyond 24 months have high and very high scores (Table 3).

Among the analyzed group, 98.3% had secondary and university education. It is noteworthy that mothers with university studies have 59.2% of high and very high scores and 16.9% of low scores.

72.4% of the mothers had received previous information (3.7% from the midwife, 8.2% from the pediatrician, 25.3% from the dentist, 62.8% by other means). In the case of the mothers who did not receive prior information, 67.8% of their responses were low and acceptable, resulting in a statistically significant relationship between lack of prior information and reduced level of knowledge (*p* < 0.05) (Tables 4, 5).

**Table 4:**
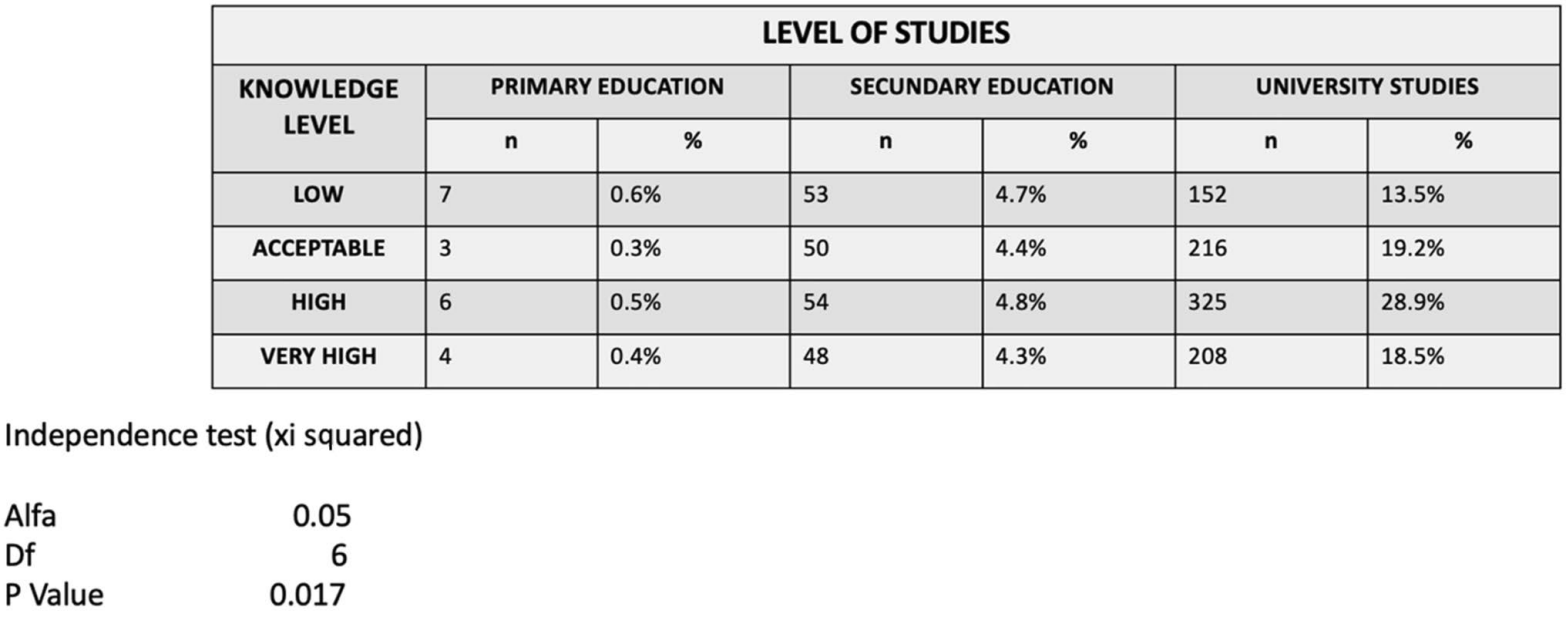
Level of knowledge according to mother’s level of studies

**Table 5:**
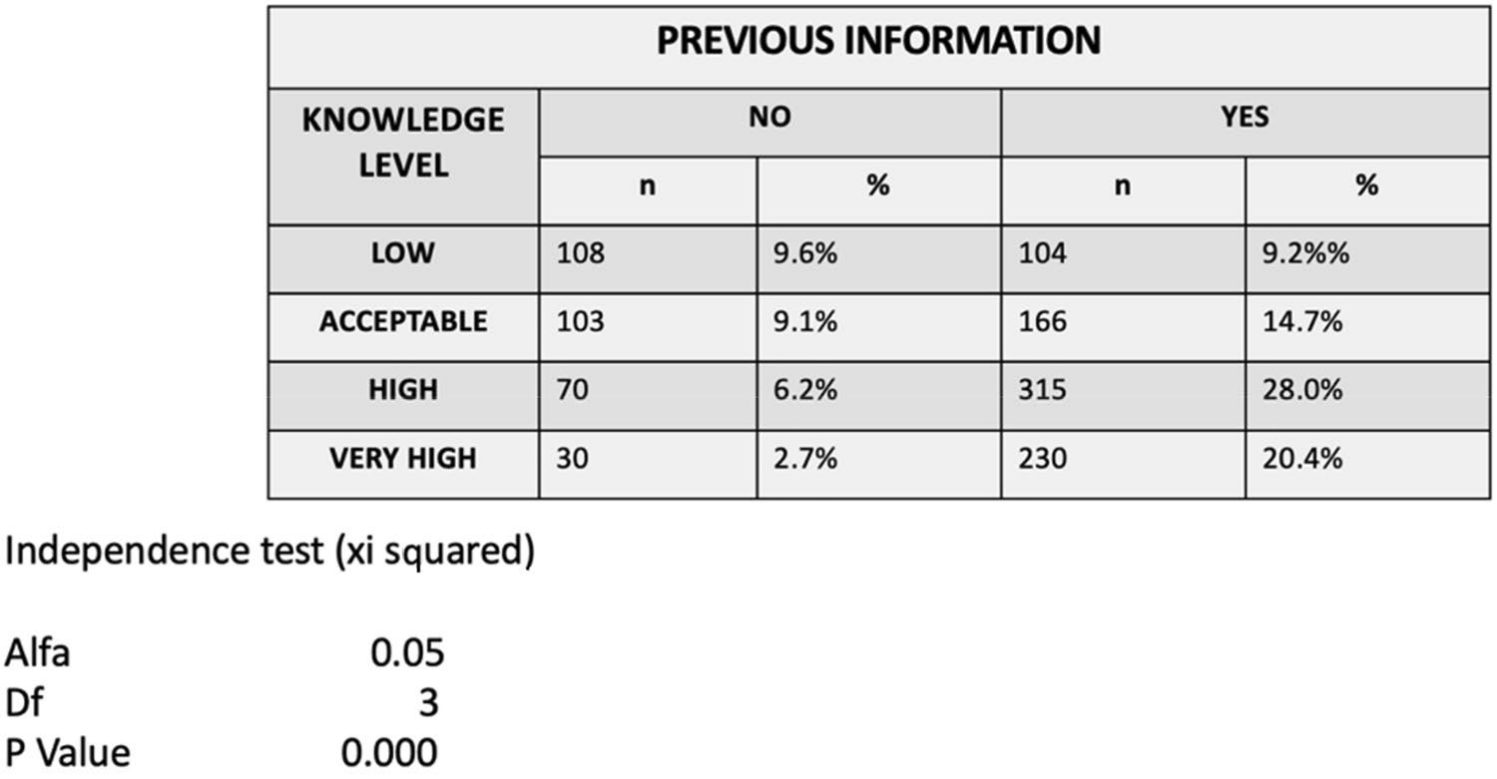
Level of mother’s knowledge according to previous information received about children’s oral health

Analyzing the results obtained according to each section of the questionnaire, we find that in the 1st block: “General knowledge on dental formation and eruption” there is a great lack of knowledge on the number of deciduous teeth, since 42.5% believe that there are 24 teeth. On the other hand, 9.6% believe that there are 32 teeth in the mouth.

In the 2nd Block: "Causes and consequences of caries", we find that 53.5% of the mothers think that the bacteria responsible for caries are present in the child's mouth from birth.

In the 3rd Block referring to "Malocclusion", in general the results obtained are high, but it should be noted that 13% of the mothers do not know the beneficial effect of breastfeeding for the development of the jaws, and 10% of those surveyed consider that digital sucking does not cause any problem.

In the 4th Block: "Pediatric dental care needs" there is a real lack of knowledge regarding the ideal period to visit the dentist; for 31% the age to visit for the first time should not be before 2 years of age, for 8% not before 6 years of age; 28% of the mothers believe that the dentist should not use fluoride in any case; 12% would take their child for a dental check-up only every 2 years, and 1% only when there is suspicion of pathology.

As for prevention, in the 5th Block we find that 48% of the mothers think that oral hygiene should begin after the eruption of the teeth, and only 4.5% after the eruption of the molars.

In addition, 20% believe that hygienic techniques should be performed without toothpaste for the teeth when the child is under 3 years of age. 13% believe that fluoride is harmful for children over 3 years of age and 15% believe that the correct amount of fluoride is 450 ppm. In the matter of brushing for 8-year-old children, 15% of them found that it should be done by the child without help, and 12% that it is only necessary to do it from time to time, as a game.

In the 6th Block, based on “Child feeding habits”, is where the best results are obtained. We found a high level of knowledge for the questions on the food-caries relationship, with 98% even recognizing “hidden sugars”.

## Discussion

The objective of this study is to assess the knowledge on infant oral health of mothers who breastfeed more than 6 months, to implement, if necessary, concrete and effective measures to promote the oral health of both mother and infant. It can also be applied to other health groups like pediatrician and midwives.

Prolonged breastfeeding is defined as breastfeeding beyond the eruption of the first primary tooth or beyond 12 months. For our study, we have taken 6 months as the reference age, since the WHO recommends exclusive breastfeeding until 6 months of age and considering that the average duration of breastfeeding in Spain is 3.2 months (Henríquez 2010; Breastfeeding Committee of the Spanish Association of Pediatrics; Cahuana et al. 2016).

According to the Breastfeeding Committee of the Spanish Association of Pediatrics, the expression "prolonged breastfeeding", although widespread, can lead to confusion, so they recommend speaking of "breastfeeding" without further adjectives, to normalize breastfeeding in children older than 1 year, since it is a maternal and child health objective (Breastfeeding Committee of the Spanish Association of Pediatrics; AEPED).

There is controversy about the possible relationship between breastfeeding and early childhood caries. While there is significant evidence of an association between breastfeeding and general health, the association with reduced dental caries is less clear. The systematic review by Riggs et al (2019) concluded that breastfeeding up to 12 months was associated with reduced dental caries, although some studies found an increase in dental caries, the trend appeared to change with breastfeeding after 12 months (Riggs et al. 2019).

The results found in the literature differ greatly because they do not distinguish whether breastfeeding is "on demand", whether it is nocturnal or daytime, the type of complementary feeding given to the child, whether oral hygiene is adequate, whether fluoride is used in adequate amounts and/or whether there is vertical bacterial transmission. Therefore, the dentist should promote breastfeeding, but emphasizing appropriate preventive measures such as: brushing according to age, the use of fluoride from the eruption of the first tooth in concentrations of no less than 1000 ppm, periodic check-ups with the pediatric dentist from approximately 6 months of age, early dental care according to the caries risk of the child, and adequate dietary control (Ramos et al. 2014; Cupé-Araujo et al. 2015; Benavente et al. 2014; Setiawatiet al. 2017; White 2008).

According to the 2020 Oral Health Survey in Spain, the socioeconomic level of the family is significantly associated with the prevalence of caries in the primary dentition in the 5–6 years age group, with the prevalence of caries (24.9% in the high level, 24.1% in the medium level and 38.3% in the low level). Therefore, it is important to assess the level of knowledge of breastfeeding mothers (Bravo et al. 2020; Bravo et al. 2015).

Chaffee et al. (2017) analyze quality of life measures related to oral health by comparing variation by socioeconomic status and caries experience. Dental caries was associated with negative child and family experiences. Socioeconomic status is a critical influence of child oral health as it is a factor in family ability to respond to dental problems (Chaffee et al. 2017).

In our study, the best scores were obtained by mothers in the age group of 30–40 years and with a higher level of education. It is noteworthy in the results that 88.3% of the surveyed mothers whose breastfeeding lasts more than 1 year of the baby's life have a high level of knowledge about children's oral health.

It is interesting to note that for mothers who have breastfed for less time (between 6 and 12 months), the level of oral health knowledge is lower compared to those who have breastfed for more than 24 months, as this may influence the acquisition of knowledge over time. Dhull et al. (2018) noted in their study that this level of knowledge and attitude of mothers towards oral health care of children between 9 and 24 months was poor, similar results to this study. The dentist is the health professional who provides more information on oral health than the pediatrician and midwife. Even so, other sources of information (magazines, internet, word of mouth, etc.) offer and give results on the level of high and very high knowledge, like to that obtained through the dentist.

Also noteworthy is the lack of information provided by both the pediatrician and the midwives. Mothers informed by both obtained the lowest scores in the test (Dhull et al. 2018; Orenuga 2005; Dickson-Swift et al. 2020).

The dentist should be integrated into the health team of pregnant mothers to promote the oral health of children from birth, since mothers during pregnancy are more receptive to acquiring healthier habits. Based on that, the prevalence of early childhood caries can be reduced, and the correct development of the growing jaws can be promoted, in addition to providing support to nursing mothers regardless of the type of breastfeeding. Similarly, Snell et al. observed that mothers accurately perceived their children's caries experience (Snell et al. 2019; American Academy of Pediatric Dentistry 2012; Sabounchi et al. 2019).

No references to surveys of breastfeeding mothers were found in the literature consulted, so we cannot make direct comparisons. There is only evidence of articles analyzing the level of oral health knowledge of parents with preschool children. Most of these studies have been carried out in countries with large sociocultural differences with respect to the current Spanish population. Likewise, the authors highlight the importance of informing mothers to improve oral health, prioritizing pregnant women because of their receptivity to habit change (Tinos and Sales-Peres 2013; Teixeira et al. 2010; Feldens et al. 2012; Delgado et al. 2016).

Current computer tools make it possible to ask a series of questions through surveys to obtain the desired information, as well as to access a very wide network of population, and to conduct studies with a larger sample size.

## Limitations

Among the limitations found in this study is that the sample population was limited to mothers who belonged to breastfeeding support associations and various midwifery groups. This work is limited to the female population, without considering that knowledge of oral health should be required in other members of the family unit. Technology is another limitation of the study, as it excludes the population that does not have computer access to the platform, nor an adequate level of education. The test sent through Google Forms^®^ does not support any option to resolve doubts about how to understand the questions. Attempts have been made to avoid response biases through the selection of the sample, to obtain higher quality information. The study population could be larger if both the response time and the sending of the questionnaire open to the general population through social networks were extended.

Finally, no similar studies on the Spanish population have been found. This limitation represents an opportunity for further research.

## Conclusions

Considering the limitations of the present study, it has been clearly shown that: the age of mothers is positively related to the highest level of oral health knowledge, the elder the mother the higher the level of knowledge.

Mothers with a longer duration of breastfeeding, beyond 24 months, have a higher degree of knowledge of oral hygiene than the rest of the respondents.

Mothers with a university education have the highest level of oral health knowledge.

There is a positively relationship between those mothers having received previous information on oral hygiene and their surveyed degree of knowledge.

## Supplementary Information

Below is the link to the electronic supplementary material.

Supplementary file 1 (DOCX 40 KB)
