# Supplementary material for: Oral health knowledge of Spanish breastfeeding mothers
Source: Eur Arch Paediatr Dent. 2022 Nov 28;24(1):139–45. doi: 10.1007/s40368-022-00769-9 (PMC9992039; doi:10.1007/s40368-022-00769-9)
Supplement: Supplementary file 1 — (DOCX 40 KB) [file 40368_2022_769_MOESM1_ESM.docx]

APPENDICES I.


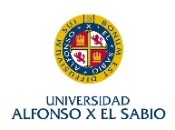


**ORAL HEALTH KNOWLEDGE OF LONG-TERM BREASTFEEDING MOTHERS**

**FIRST BLOCK**: general knowledge of tooth formation and eruption

1.-During pregnancy, the baby gets calcium to form its teeth:

- From the calcium in the mother's teeth.
- From the calcium in the mother's bones.
- From a balanced diet of the mother.

2.-The baby teeth begin to form:

- From the sixth week of pregnancy.
- From the birth of the baby.
- From six months of age.

3.-At what approximate age does the last milk tooth erupt?

- By 12 months.
- By 18 months.
- By 24 months.

4.- How many teeth does the milk dentition have?

- 32 teeth.
- 24 teeth.
- 20 teeth.

**SECOND BLOCK**: Causes and consequences of tooth decay

5.-Cavities in the milk teeth can produce:

- Severe pain.
- Higher caries rate in permanent teeth.
- Infections.
- All of the above.

6.-The child's caries has a colour:

- Black or brown.
- Dull whitish or yellowish.
- All of the above are correct.

7.-With regard to caries, it is true that:

- It is a genetic disease.
- Its only cause is the intake of sugars.
- It is a chronic disease that depends on many factors.

8.-The bacteria that cause caries are transmitted:

- For spoiled food.
- From parents to children by kissing on the mouth, sharing spoons, etc.
- They are present in the baby's mouth from birth.

**THIRD BLOCK**: Causes of malocclusion

9.- Is breastfeeding favourable for the development of the baby's mouth?

- Yes, sucking on the breast helps your baby's mouth to grow properly.
- No. The advantages of breastfeeding are different.
- No, it is not favourable because it causes narrowing of the palate.

10.-The use of dummies:

- It is not recommended.
- If used, it has to be removed at 18-24 months of age.
- May cause malocclusions.
- All of the above are correct.

11.- To avoid an incorrect development of the child's mouth (malocclusion), it is beneficial:

- That the baby teeth are not lost prematurely.
- Use a large soother to form the palate correctly.
- Breathe through the mouth to widen the palate.

12.-Thumb sucking:

- Long-term effects on the child's bite.
- It is a habit that is easily eliminated.
- This is common and unimportant in young children.

**FOURTH BLOCK**: paediatric dentistry care needs

13.-At what age should children go to their dentist (paediatric dentist)?

- From the age of two.
- Before the age of one year.
- At the age of six, with the eruption of the permanent teeth.

14.- If there is a risk of caries in the child, the paediatric dentist may:

- Prescribe fluoride tablets.
- Use topical fluoride in varnishes, gels etc.
- Fluoride is completely contraindicated in young children.

15.- If a child knocks out a baby tooth, it is important:

- Do not brush your teeth for two days so that it does not move.
- See a paediatric dentist as soon as possible.
- If it has fallen down, put it back in place as quickly as possible.

16.-Children should see their dentist (paediatric dentist):

- At least every six months for a dental check-up.
- Every twelve months.
- Only when caries or other pathologies are present.

**FIFTH BLOCK**: preventive and hygienic habits

17.-When should you start cleaning your child's mouth?

- Before the eruption of the teeth with a wet gauze.
- From the eruption of the molars (18-24 months).
- From the eruption of the first teeth (6 months).

18.-Toothpaste for the hygiene of children from 6 months to 3 years of age:

- Must have at least 1,000 parts per million fluorine.
- It is harmful if it contains fluoride.
- It is not advisable to use toothpaste at this age.

19.-Toothpaste for the hygiene of children from 3 years of age:

- Must have 450 parts per million fluorine.
- It should have 1000 to 1450 parts per million fluorine.
- It is harmful if it contains fluoride.

20.- Regarding dental cleaning from the first year of age:

- It is advisable for the child to brush his or her own teeth.
- It is only necessary from time to time, as a game, to get into the habit.
- This should be done by parents up to the age of 7-8 years.

**SIXTH BLOCK**: Eating habits

21.-In order to prevent infant caries it is recommended:

- Do not offer sugars before two years of age.
- Do not drink soft drinks.
- Do not eat buns and biscuits.
- All of the above are correct.

22.- In order to avoid infantile caries it is advisable:

- Eating very frequent meals.
- Avoid snacking.
- If sweets are taken between main meals.

23.- It is advisable to avoid the consumption of "hidden sugars" present for example in:

- Bags of crisps and savoury snacks.
- Sliced bread.
- Industrial juices and soft drinks.
- All of the above are correct.

24.- What types of food are the most suitable for the correct development of the jaws?

- The soft ones, so that the surfaces of the teeth are not abraded.
- The hard and fibrous ones to increase chewing.
- The crushed ones, as swallowing them correctly positions the jaw.
